# Supplementary material for: The Pathogen-Induced MATE Gene TaPIMA1 Is Required for Defense Responses to Rhizoctonia cerealis in Wheat
Source: Int J Mol Sci. 2022 Mar 21;23(6):3377. doi: 10.3390/ijms23063377 (PMC8950252; doi:10.3390/ijms23063377)
Supplement: Supplementary file 1 [file ijms-23-03377-s001.zip › ijms-1616333-supplementary.pdf]

```
TaPIMA1    ..MAGGDEQEQEHGAASCRLESIL........TDTSA.PLAERAW.AAGAVELRLLSRLAAPAVVVYMINYVMSMSTQIF     68
AtTT12     .MSSTETYEPLLTRLHSDSQITERSSPEIEEFLRRRGSTVTPRWWLKLAVWESKLLWTLSGASIVVSVLNYMLSFVTVMF     79
GhTT12     MGSAAPEYQPLLLGLDSHSRIPDLSSVAIEEFLQHR..PIALRWWPRLVAWESRLLWLLSGSSIVLSIFNYMLSFVTLMF     78
Consensus                  s                         r w      e  ll  l     v    ny  s  t  f

TaPIMA1    SGHLGNLELAAASLGNTGVQTFAYGLLLGMGSAVETLCGQAYGAHKYDMLGIYLQRSVILLGLTGIPLAVMYALSEPLLL    148
AtTT12     TGHLGSLQLAGASIATVGIQGLAYGIMLGMASAVQTVCGQAYGARQYSSMGIICQRAMVLHLAAAVFLTFLYWYSGPILK    159
GhTT12     TGHLGALELAGASIASVGIQGLAYGIMLGMASAVQTVCGQAYGAKQYSAMGIICQRAIILHLGAAVLLTFLYWFSGDVLQ    158
Consensus   ghlg l la as    g q  ayg  lgm sav t cgqayga  y   gi  qr   l       l   y  s   l

TaPIMA1    LMGQSLEIAHATSIFVYGLIPQIFAYAVNFPIQKFLQAQSIVLPSAYISTATLVLHVLMSWVLMYKVGLGLLGASLVLSV    228
AtTT12     TMGQSVAIAHEGQIFARGMIPQIYAFALACPMQRFLQAQNIVNPLAYMSLGVFLLHTLLTWLVTNVLDFGLLGAALILSF    239
GhTT12     AIGQTESIAQQGQVFSRGLIPQIYAFAISCPMQRFLQAQNIVNPLAFMSIGIFLVHVLLTWLVVNVLGCGLLGAALTLSL    238
Consensus    gq   ia     f  g ipqi a a   p q flqaq iv p a  s      h l  w        gllga l ls

TaPIMA1    SWWIIVAAQFVYIVVSPTCRHTWTGLSWQAFSGLPSFFKLSAASAVMLCLEAWYFQVLVIIAGLLPNPEIALDSLSICMT    308
AtTT12     SWWLLVAVNGMYILMSPNCKETWTGFSTRAFRGIWPYFKLTVASAVMLCLEIWYNQGLVIISGLLSNPTISLDAISICMY    319
GhTT12     SWWFLVVINGLYIVLSPSCKETWSGLSFRAFTGIWPYFKLTVASAVMLCLEIWYNQGLVLISGLLSNPTIALDSISICMN    318
Consensus  sww  v     yi  sp c  tw g s  af g    fkl  asavmlcle wy q lv i gll np i ld  sicm

TaPIMA1    IYGWVFMISVGFNAAASVRVSNELGAGNPKSAFFSVWVVTGISATISTILAIVILCLRNHISYLFTDGEAVSDAVADLCP    388
AtTT12     YLNWDMQFMLGLSAAISVRVSNELGAGNPRVAMLSVVVVNITTVLISSVLCVIVLVFRVGLSKAFTSDAEVIAAVSDLFP    399
GhTT12     YLNWDMQFMLGLSAAASVRVSNELGAGHPRVAKFSVFVVNGTSILISIVFSAIVLIFRVGLSKAFTSDSEVIEAVSDLTP    398
Consensus     w      g  aa svrvsnelgag p  a  sv vv      is       l  r   s ft     v  av dl p

TaPIMA1    FLAVTLVLGGIQPVLTGVAVGCGWQQFVAYVNVGSYYIVGVPLGVVLGFFFNLGAKGIWGGLIGGTALQTAILLWVTIRT    468
AtTT12     LLAVSIFLNGIQPILSGVAIGSGWQAVVAYVNLVTYYVIGLPIGCVLGFKTSLGVAGIWWGMIAGVILQTLTLIVLTLKT    479
GhTT12     LLAISVFLNGIQPILSGVAIGSGWQAIVAYVNLATYYIIGLPIGCVLGFKTSLGVAGIWWGMIIGVLLQTATLVVLTATT    478
Consensus   la    l giqp l gva g gwq  vayvn   yy  g p g vlgf   lg  giw g i g  lqt  l   t  t

TaPIMA1    DWTKEVEEAHKRLNKWDGKKDPLLTGFKEN                                                      498
AtTT12     NWTSEVENAAQRVKTSATENQEMANAGV..                                                      507
GhTT12     NWNKEVEKAADRLKKSANEEQT........                                                      500
Consensus   w  eve a  r
```

Figure S1. Multiple amino acid sequences alignment of TaPIMA1, AtTT12 and GhTT12.

A *TaPIMA1* VIGS fragment

GCCCTGGATTCCCTCTCTATATGTATGACGATTTATGGTTGGGTGTTCATGATCT
CTGTTGGGTTCAATGCCGCTGCAAGTGTGAGAGTGAGCAACGAGCTTGGCGCC
GGCAACCCAAAGTCTGCATTTTTCTCTGTGTGGGTCGTGACTGGAATCTCTGCG
ACAATCTCTACCATCCTTGCTATTGTGATCCTCTGCCTTCGCAACCACATTAGC

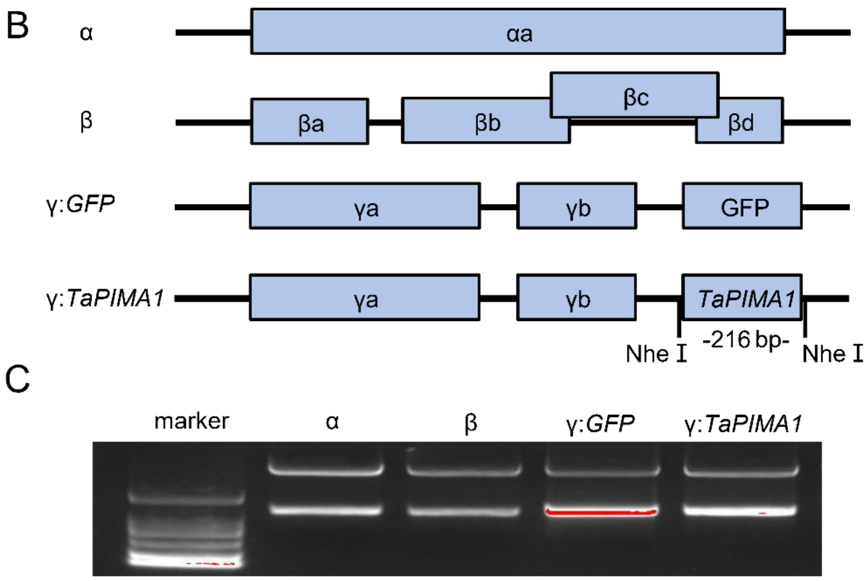

Figure S2. (A) The VIGS fragment of *TaPIMA1*. (B) A simplified schema of BSMV RNAs construct. (C) The transcription of BSMV *in vitro*. The red area represents image overexposure.

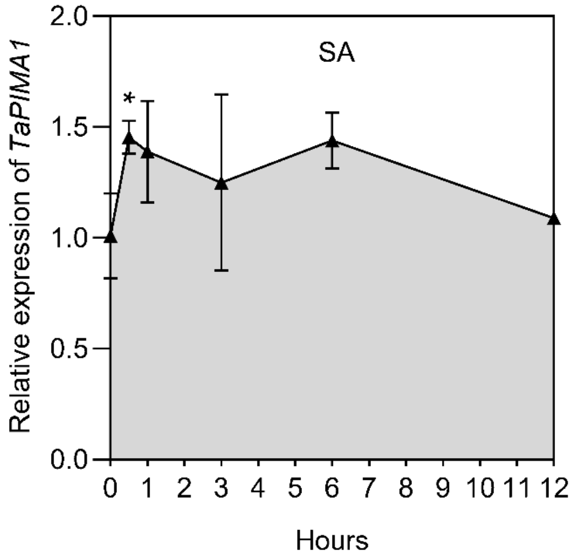

Figure S3. The transcriptional profiles of *TaPIMA1* in SA treated CI12633 plants. The plants were sprayed with 0.1 mM SA and 0.1% tween-20 (control) at four-leaf stage, respectively. Error bars indicates standard deviation. *TaActin* was used as internal control.

Table S1 Primers were used in this study.

Primer name	Sequence (5´–3´)	Use
TaPIMA1-F1	5'-CAGTGCAGTGGAGACCCT-3'	PCR for cDNA amplification
TaPIMA1-R1	5'-TGTGCCGTTGAGATGGA-3'	PCR for cDNA amplification
TaPIMA1-F2	5'-ATGCTCGGAATCTACCTACA-3'	PCR for cDNA amplification
TaPIMA1-R2	5'-GTGCCTCCTCTACCTCTTT-3'	PCR for cDNA amplification
TaPIMA1-VIGS-F	5'-TATGCTAGCGCCCTGGATTCCCTCTCT-3'	VIGS
TaPIMA1-VIGS-R	5'-ATTGCTAGCGCTAATGTGGTTGCGAAGG-3'	VIGS
TaPIMA1-sub-F	5'-CCAAGCTTATGGCTGGCGGGGACGAGCA-3'	subcellular localization
TaPIMA1-sub-R	5'- CACTGCAGGTTATTTTCCTTGAATCCTG -3'	subcellular localization
TaActinRTF	5'-GGAATCCATGAGACCACCTAC-3'	RT-qPCR
TaActinRTR	5'-GACCCAGACAACTCGCAAC-3'	RT-qPCR
BSMV-CPF	5'-TGACTGCTAAGGGTGGAGGA-3'	RT-PCR
BSMV-CPR	5'-CGGTTGAACATCACGAAGAGT-3'	RT-PCR
DefensinF	5'-ATGTCCGTGCCTTTTGCTA-3'	RT-qPCR
DefensinR	5'-CCAAACTACCGAGTCCCCG-3'	RT-qPCR
Chitinase3-F	5'-CCCACCCTAACCTGAGCATC-3'	RT-qPCR
Chitinase3-R	5'-ACTGGTTGATCATGGCGGAG-3'	RT-qPCR
PR1.2-QF	5′- CGTCTTCATCACCTGCAACTA-3′	RT-qPCR
PR1.2-QR	5′- CAAACATAAACACACGCACGTA-3′	RT-qPCR
PR10F	5'-CGTGGAGGTAAACGATGAG-3'	RT-qPCR
PR10R	5'-GCTAAGTGTCCGGGGTAAT-3'	RT-qPCR

The underline represents the enzyme digestion site.

Table S2. The MATE transporters were used in phylogenetic tree analysis.

Protein name	Organism	Accession numbers	Functions	Reference
SbMATE	*Sorghum bicolor*	Sb03g043890	aluminum tolerance	[1]
AtADS1	*Arabidopsis thaliana*	AT4G29140	disease resistance	[2]
AhFRDL1	*Arachis hypogaea*	AXX83001	Fe-translocation/aluminum tolerance	[3]
VvAM1	*Vitis vinifera*	NP-001268037	anthocyanidins transport	[4]
AtFRD3	*Arabidopsis thaliana*	AT3G08040	iron and zinc homeostasis	[5]
AtMATE	*Arabidopsis thaliana*	AT1G51340	aluminum tolerance	[6]
BdMATE	*Brachypodium distachyon*	BRADI_1g69770	aluminum tolerance	[7]
AtDTX1	*Arabidopsis thaliana*	AT2G04040	detoxification of heavy metals	[8]
AtDTX18	*Arabidopsis thaliana*	NP_188997	detoxification	[9]
AtEDS5	*Arabidopsis thaliana*	NP_195614.2	disease resistance	[10-13]
AtEDS5H	*Arabidopsis thaliana*	NP_565509.4	disease resistance	[14]
GhTT12	*Gossypium hirsutum*	AGW32085	transport of proanthocyanin	[15]
HvMATE	*Hordeum vulgare*	XP_044982392.1	aluminum tolerance	[16]
MtMATE2	*Medicago truncatula*	ADV04045.1	aluminium tolerance	[17]
OsFRDL1	*Oryza sativa*	Os03g08900	Fe-translocation	[18]
SiMATE1	*Setaria italica*	XP_004985179.1	aluminium tolerance	[16]
TaMATE1B	*Triticum aestivum*	AFZ61900.1	aluminum tolerance	[19]
TaMATE2	*Triticum aestivum*	AWW07047.1	aluminium tolerance	[20]
TuMATE1	*Triticum urartu*	EMS52414.1	aluminium tolerance	[16]
TaMATE2	*Triticum aestivum*	AWW07047.1	aluminium tolerance	[20]
HvAACT1	*Hordeum vulgare*	ANN88347	aluminium tolerance	[21]
OsMATE1	*Oryza sativa*	ABF94377.1	disease resistance	[22]
OsMATE2	*Oryza sativa*	AHD46250.1	disease resistance	[22]
TraesCS2B01G296000	*Triticum aestivum*	KAF7008897.1	disease resistance	[23]
TaPIMA1	*Triticum aestivum*	TraesCS3B02G563500.1	disease resistance	

Table S3. The infection types (ITs) and disease indexes (DIs) of the BSMV-infected CI12633 plants.

Index	Batch 1		Batch 2	
	BSMV:GFP	BSMV:TaPIMA1	BSMV:GFP	BSMV:TaPIMA1
ITs	1.25±0.64	2.29±1.44**	1.27±0.61	2.67±1.24**
DIs	25.00±12.72	45.88±28.95**	25.41±12.16	53.33±24.79**

The significant differences between BSMV:GFP infected and BSMV:TaPIMA1 infected plants were determined by one-way ANOVA (*<0.05, ***P*<0.01).

Table S4. Raw data of qRT-PCR.

Materials	Actin' C_T values			Target genes' C_T values			Target genes
	1	2	3	1	2	3	
CI-leave	20.198	20.201	20.454	28.048	28.102	27.927	*TaPIMA1*
CI-spike	18.965	19.078	19.050	32.667	32.988	32.879	*TaPIMA1*
CI-stem	20.394	21.268	20.231	28.725	29.872	29.190	*TaPIMA1*
CI-root	18.727	18.080	18.041	29.452	29.179	29.522	*TaPIMA1*
W6-mock	21.865	22.587	22.435	27.934	27.619	28.336	*TaPIMA1*
W6-1 d	19.289	19.136	19.319	24.556	24.502	24.381	*TaPIMA1*
W6-4 d	19.416	19.741	19.761	24.914	24.743	24.994	*TaPIMA1*
W6-10 d	18.126	18.226	18.347	24.401	24.433	24.345	*TaPIMA1*
CI-BSMV-GFP-1	24.515	26.147	25.455	21.709	22.383	21.771	*RcActin*
CI-BSMV-GFP-2	24.991	24.497	24.787	27.261	27.237	26.817	*RcActin*
CI-BSMV-GFP-3	23.380	23.097	22.495	22.669	22.267	21.667	*RcActin*
CI-BSMV-TaPIMA1-1	23.654	22.912	23.084	21.088	20.398	20.752	*RcActin*
CI-BSMV-TaPIMA1-2	23.994	24.115	23.923	20.895	20.909	20.027	*RcActin*
CI-BSMV-TaPIMA1-3	25.303	25.378	25.591	21.294	21.465	21.799	*RcActin*
CI-BSMV-GFP-1	25.396	25.703	25.754	30.306	30.470	30.448	*TaPIMA1*
CI-BSMV-GFP-2	26.153	26.446	26.533	29.767	30.196	30.907	*TaPIMA1*
CI-BSMV-GFP-3	25.570	25.601	25.688	31.023	31.002	30.698	*TaPIMA1*
CI-BSMV-TaPIMA1-1	25.948	26.289	26.154	32.682	32.611	33.133	*TaPIMA1*
CI-BSMV-TaPIMA1-2	25.628	25.639	26.071	31.410	31.531	31.602	*TaPIMA1*
CI-BSMV-TaPIMA1-3	25.478	25.309	26.064	32.063	31.978	32.485	*TaPIMA1*
CI-BSMV-GFP-1	23.320	23.290	23.394	19.847	19.843	19.872	*PR1.2*
CI-BSMV-GFP-2	22.890	23.178	23.214	21.593	21.629	21.616	*PR1.2*
CI-BSMV-GFP-3	23.137	23.216	23.275	22.222	21.912	22.226	*PR1.2*
CI-BSMV-TaPIMA1-1	23.170	23.200	23.327	23.467	23.482	23.501	*PR1.2*
CI-BSMV-TaPIMA1-2	23.322	23.307	23.309	23.715	23.664	23.376	*PR1.2*
CI-BSMV-TaPIMA1-3	23.035	23.376	23.478	26.210	26.268	26.159	*PR1.2*
CI-BSMV-GFP-1	27.286	27.485	27.416	30.143	30.020	30.288	*chitinase3*
CI-BSMV-GFP-2	28.565	28.501	28.572	31.132	31.099	30.486	*chitinase3*
CI-BSMV-GFP-3	27.321	27.698	27.678	31.040	31.145	30.567	*chitinase3*
CI-BSMV-TaPIMA1-1	27.811	27.694	27.720	32.942	32.001	32.010	*chitinase3*
CI-BSMV-TaPIMA1-2	28.205	28.346	28.297	32.439	32.228	32.151	*chitinase3*
CI-BSMV-TaPIMA1-3	28.825	28.594	28.913	32.717	33.149	32.754	*chitinase3*
CI-BSMV-GFP-1	28.203	28.258	28.139	29.890	30.173	29.934	*defensin*
CI-BSMV-GFP-2	28.285	28.390	28.129	30.248	30.033	29.953	*defensin*
CI-BSMV-TaPIMA1-1	28.444	28.409	28.496	32.742	32.152	32.334	*defensin*
CI-BSMV-TaPIMA1-2	29.059	28.643	28.969	32.619	33.164	33.367	*defensin*
CI-BSMV-TaPIMA1-3	29.261	29.490	29.477	33.238	32.800	33.110	*defensin*
CI-BSMV-GFP-1	22.912	22.855	23.154	20.179	20.401	20.346	*PR10*
CI-BSMV-GFP-2	22.921	22.786	22.877	19.443	19.387	19.614	*PR10*
CI-BSMV-GFP-3	23.848	23.909	23.937	20.917	20.969	20.961	*PR10*
CI-BSMV-TaPIMA1-1	21.749	21.812	21.816	19.724	19.776	19.680	*PR10*

Table S4. *Cont.*

Materials	Actin' C_T values			Target genes' C_T values			Target genes
	1	2	3	1	2	3	
CI-BSMV-TaPIMA1-2	22.233	21.964	21.974	19.779	19.794	19.883	*PR10*
CI-BSMV-TaPIMA1-3	21.031	20.868	20.997	19.745	19.750	19.855	*PR10*
CI12633-JA-0	24.239	24.158	24.137	26.754	26.890	26.589	*TaPIMA1*
CI12633-JA-0.5 h	21.919	22.149	22.340	23.635	23.423	23.290	*TaPIMA1*
CI12633-JA-1 h	22.249	22.463	22.561	24.520	24.352	24.707	*TaPIMA1*
CI12633-JA-3 h	22.603	22.300	22.147	24.547	24.534	24.060	*TaPIMA1*
CI12633-JA-6 h	22.452	22.904	22.798	24.767	24.859	24.128	*TaPIMA1*
CI12633-JA-12 h	23.308	23.713	23.036	26.627	26.603	26.510	*TaPIMA1*
CI12633-H_2O_2-0	22.225	21.886	21.942	34.218	34.469	33.876	*TaPIMA1*
CI12633-H_2O_2-0.5 h	24.890	24.838	25.111	35.166	35.914	35.321	*TaPIMA1*
CI12633-H_2O_2-1 h	21.864	21.979	22.197	31.811	31.770	31.342	*TaPIMA1*
CI12633-H_2O_2-3 h	22.130	22.322	22.396	30.086	29.831	29.519	*TaPIMA1*
CI12633-H_2O_2-6 h	21.256	21.371	21.535	30.068	30.160	29.707	*TaPIMA1*
CI12633-H_2O_2-12 h	21.833	21.926	21.737	30.063	30.229	29.663	*TaPIMA1*
CI12633-SA-0	22.975	23.549	23.193	30.954	31.137	31.145	*TaPIMA1*
CI12633-SA-0.5 h	23.894	23.756	23.534	31.191	30.947	31.169	*TaPIMA1*
CI12633-SA-1 h	23.461	23.498	23.356	30.894	30.613	30.933	*TaPIMA1*
CI12633-SA-3 h	23.688	23.785	23.668	30.859	31.616	31.037	*TaPIMA1*
CI12633-SA-6 h	23.333	23.536	23.634	30.722	30.700	30.811	*TaPIMA1*
CI12633-SA-12 h	26.663	26.726	26.945	34.298	34.413	34.554	*TaPIMA1*

Reference

1. Melo, J.O.; Martins, L.G.C.; Barros, B.A.; Pimenta, M.R.; Lana, U.G.P.; Duarte, C.E.M.; Pastina, M.M.; Guimaraes, C.T.; Schaffert, R.E.; Kochian, L.V., et al. Repeat variants for the SbMATE transporter protect sorghum roots from aluminum toxicity by transcriptional interplay in cis and trans. *Proc. Natl. Acad. Sci. U. S. A.* **2019,** 116, 313-318.
2. Sun, X.L.; Gilroy, E.M.; Chini, A.; Nurmberg, P.L.; Hein, I.; Lacomme, C.; Birch, P.R.J.; Hussain, A.; Yun, B.W.; Loake, G.J. *ADS1* encodes a MATE-transporter that negatively regulates plant disease resistance. *New Phytol.* **2011,** 192, 471-482.
3. Qiu, W.; Wang, N.; Dai, J.; Wang, T.; Kochian, L.V.; Liu, J.; Zuo, Y. AhFRDL1-mediated citrate secretion contributes to adaptation to iron deficiency and aluminum stress in peanuts. *J. Exp. Bot.* **2019,** 70, 2873-2886.
4. Gomez, C.; Terrier, N.; Torregrosa, L.; Vialet, S.; Fournier-Level, A.; Verries, C.; Souquet, J.M.; Mazauric, J.P.; Klein, M.; Cheynier, V., et al. Grapevine MATE-type proteins act as vacuolar H^+-dependent acylated anthocyanin transporters. *Plant Physiol.* **2009,** 150, 402-415.
5. Pineau, C.; Loubet, S.; Lefoulon, C.; Chalies, C.; Fizames, C.; Lacombe, B.; Ferrand, M.; Loudet, O.; Berthomieu, P.; Richard, O. Natural variation at the *FRD3* MATE transporter locus reveals cross-talk between Fe homeostasis and Zn tolerance in *Arabidopsis thaliana*. *PLoS Genet.* **2012,** 8, e1003120.
6. Liu, J.; Luo, X.; Shaff, J.; Liang, C.; Jia, X.; Li, Z.; Magalhaes, J.; Kochian, L.V. A promoter-swap strategy between the *AtALMT* and *AtMATE* genes increased *Arabidopsis* aluminum resistance and improved carbon-use efficiency for aluminum resistance. *Plant J.* **2012,** 71, 327-337.
7. Ribeiro, A.P.; de Souza, W.R.; Martins, P.K.; Vinecky, F.; Duarte, K.E.; Basso, M.F.; da Cunha, B.A.D.B.; Campanha, R.B.; de Oliveira, P.A.; Centeno, D.C., et al. Overexpression of *BdMATE* gene improves aluminum tolerance in *Setaria viridis*. *Front. Plant Sci.* **2017,** 8, 865-865.
8. Li, L.; He, Z.; Pandey, G.K.; Tsuchiya, T.; Luan, S. Functional cloning and characterization of a plant efflux carrier for multidrug and heavy metal detoxification. *J. Biol. Chem.* **2002,** 277, 5360-5368.
9. Li, J.; Meng, Y.; Zhang, K.; Li, Q.; Li, S.; Xu, B.; Georgiev, M.I.; Zhou, M. Jasmonic acid-responsive RRTF1 transcription factor controls *DTX18* gene expression in hydroxycinnamic acid amide secretion. *Plant Physiol.* **2021,** 185, 369-384.
10. Ishihara, T.; Sekine, K.T.; Hase, S.; Kanayama, Y.; Seo, S.; Ohashi, Y.; Kusano, T.; Shibata, D.; Shah, J.; Takahashi, H. Overexpression of the *Arabidopsis thaliana EDS5* gene enhances resistance to viruses. *Plant Biology* **2008,** 10, 451-461.
11. Serrano, M.; Wang, B.; Aryal, B.; Garcion, C.; Abou-Mansour, E.; Heck, S.; Geisler, M.; Mauch, F.; Nawrath, C.; Métraux, J.P. Export of salicylic acid from the chloroplast requires the multidrug and toxin extrusion-like transporter EDS5. *Plant Physiol.* **2013,** 162, 1815-1821.
12. Yamasaki, K.; Motomura, Y.; Yagi, Y.; Nomura, H.; Kikuchi, S.; Nakai, M.; Shiina, T. Chloroplast envelope localization of EDS5, an essential factor for salicylic acid biosynthesis in *Arabidopsis thaliana*. *Plant Signal Behav.* **2013,** 8.
13. Carviel, J.L.; Wilson, D.C.; Isaacs, M.; Carella, P.; Catana, V.; Golding, B.; Weretilnyk, E.A.; Cameron, R.K. Investigation of intercellular salicylic acid accumulation during compatible and incompatible *Arabidopsis-Pseudomonas syringae* interactions using a fast neutron-generated mutant allele of *EDS5* identified by genetic mapping and whole-genome sequencing. *PloS ONE* **2014,** 9, e88608.

14. Parinthawong, N.; Cottier, S.; Buchala, A.; Nawrath, C.; Métraux, J.P. Localization and expression of EDS5H a homologue of the SA transporter EDS5. *BMC Plant Biol* **2015,** 15, 135.
15. Gao, J.S.; Wu, N.; Shen, Z.L.; Lv, K.; Qian, S.H.; Guo, N.; Sun, X.; Cai, Y.P.; Lin, Y. Molecular cloning, expression analysis and subcellular localization of a *TRANSPARENT TESTA 12* ortholog in brown cotton (*Gossypium hirsutum* L.). *Gene* **2016,** 576, 763-769.
16. Devanna, B.N.; Jaswal, R.; Singh, P.K.; Kapoor, R.; Jain, P.; Kumar, G.; Sharma, Y.; Samantaray, S.; Sharma, T.R. Role of transporters in plant disease resistance. *Physiol. Plantarum* **2021,** 171, 849-867.
17. Zhao, J.; Huhman, D.; Shadle, G.; He, X.Z.; Sumner, L.W.; Tang, Y.; Dixon, R.A. MATE2 mediates vacuolar sequestration of flavonoid glycosides and glycoside malonates in Medicago truncatula. *Plant Cell* **2011,** 23, 1536-1555.
18. Yokosho, K.; Yamaji, N.; Ueno, D.; Mitani, N.; Ma, J.F. OsFRDL1 is a citrate transporter required for efficient translocation of iron in rice. *Plant physiol.* **2009,** 149, 297-305.
19. Tovkach, A.; Ryan, P.R.; Richardson, A.E.; Lewis, D.C.; Rathjen, T.M.; Ramesh, S.; Tyerman, S.D.; Delhaize, E. Transposon-mediated alteration of *TaMATE1B* expression in wheat confers constitutive citrate efflux from root apices. *Plant Physiol.* **2013,** 161, 880-892.
20. Garcia-Oliveira, A.L.; Benito, C.; Guedes-Pinto, H.; Martins-Lopes, P. Molecular cloning of *TaMATE2* homoeologues potentially related to aluminium tolerance in bread wheat (*Triticum aestivum* L.). *Plant Biol.* **2018,** 20, 817-824.
21. FERREIRA; Jéssica; Rosset. Is a non-synonymous SNP in the *HvAACT1* coding region associated with acidic soil tolerance in barley? *Genet. Mol. Biol.* **2017,** 40.
22. Tiwari, M.; Sharma, D.; Singh, M.; Tripathi, R.D.; Trivedi, P.K. Expression of *OsMATE1 and OsMATE2* alters development, stress responses and pathogen susceptibility in Arabidopsis. *Sci. Rep.* **2014,** 4.
23. Pan, Y.; Liu, Z.; Rocheleau, H.; Fauteux, F.; Wang, Y.; Mccartney, C.; Ouellet, T. Transcriptome dynamics associated with resistance and susceptibility against fusarium head blight in four wheat genotypes. *Bmc Genomics* **2018,** 19, 642.
